# Rad5 participates in lesion bypass through its Rev1-binding and ubiquitin ligase domains, but not through its helicase function

**DOI:** 10.3389/fmolb.2022.1062027

**Published:** 2022-12-01

**Authors:** Katarzyna H. Masłowska, Vincent Pagès

**Affiliations:** Cancer Research Center of Marseille: Team DNA Damage and Genome Instability CNRS, Aix Marseille University, Inserm, Institut Paoli-Calmettes, Marseille, France

**Keywords:** DNA damage, DNA damage response (DDR), translesion synthesis (TLS), homologous recombination (HR), Rad5 (HTLF), Saccharomyces cerevisiae

## Abstract

DNA Damage Tolerance (DDT) functions to bypass replication-blocking lesions and is divided into two distinct pathways: error-prone Translesion Synthesis (TLS) and error-free Damage Avoidance (DA). Rad5 is a multifunctional protein that is involved in these DDT processes. *Saccharomyces cerevisiae* Rad5 contains three well defined domains: a RING domain that promotes PCNA polyubiquitination, a ssDNA-dependent ATPase/helicase domain, and a Rev1-binding domain. Both the RING domain and the ATPase/helicase domain are conserved in human Rad5 ortholog HLTF. In this study we used domain-specific mutants to address the contribution of each of the Rad5 domains to the lesion tolerance. We demonstrate that the two critical functions of Rad5 during DNA damage tolerance are the activation of template switching through polyubiquitination of PCNA and the recruitment of TLS polymerases, and that loss of one of those functions can be compensated by increased usage of the other. We also show that, unlike previously suggested, the helicase activity does not play any role in lesion tolerance.

## Introduction

The DNA of every living cell is constantly threatened by various damaging agents. Despite the efficient action of DNA repair mechanisms, some damage may persist long enough to be present during replication, blocking the replicative polymerases, which threatens genome stability (Friedberg et al., 2006). Therefore, to complete replication, cells need to tolerate the encountered DNA damage. There are two distinct DNA Damage Tolerance (DDT) mechanisms: i) error-prone Translesion Synthesis (TLS), employing specialized low-fidelity DNA polymerases able to insert nucleotides opposite the lesion (Sale et al., 2012); ii) Damage Avoidance (DA), a generally error-free pathway that relies on homologous recombination (HR) to retrieve the genetic information from the non-damaged sister chromatid (Branzei, 2011) (also reviewed in (Waters et al., 2009; Branzei and Szakal, 2016)). The balance between error-prone TLS and error-free DA defines the level of mutagenesis during lesion bypass. However, the current understanding of the precise molecular mechanisms regulating the process of DNA Damage Tolerance is far from complete.

In eukaryotes, lesion tolerance is controlled by the ubiquitination of proliferating cell nuclear antigen (PCNA) [reviewed in (Andersen et al., 2008; Che et al., 2021)]. PCNA monoubiquitination by Rad6 and Rad18 promotes the recruitment of TLS polymerases. Extending this modification to polyubiquitination by Mms2/Ubc13 and Rad5, enables the recombination-mediated mechanisms (Hoege et al., 2002).

Rad5 is a large multifunctional protein that contains both ubiquitin ligase and ssDNA-dependent ATPase activities (Unk et al., 2010). These overlapping domains and functions are shared with its human orthologs HLTF and SHPRH (Unk et al., 2006; Unk et al., 2008). Thus, these shared features may be of physiological importance.

**FIGURE 1:**
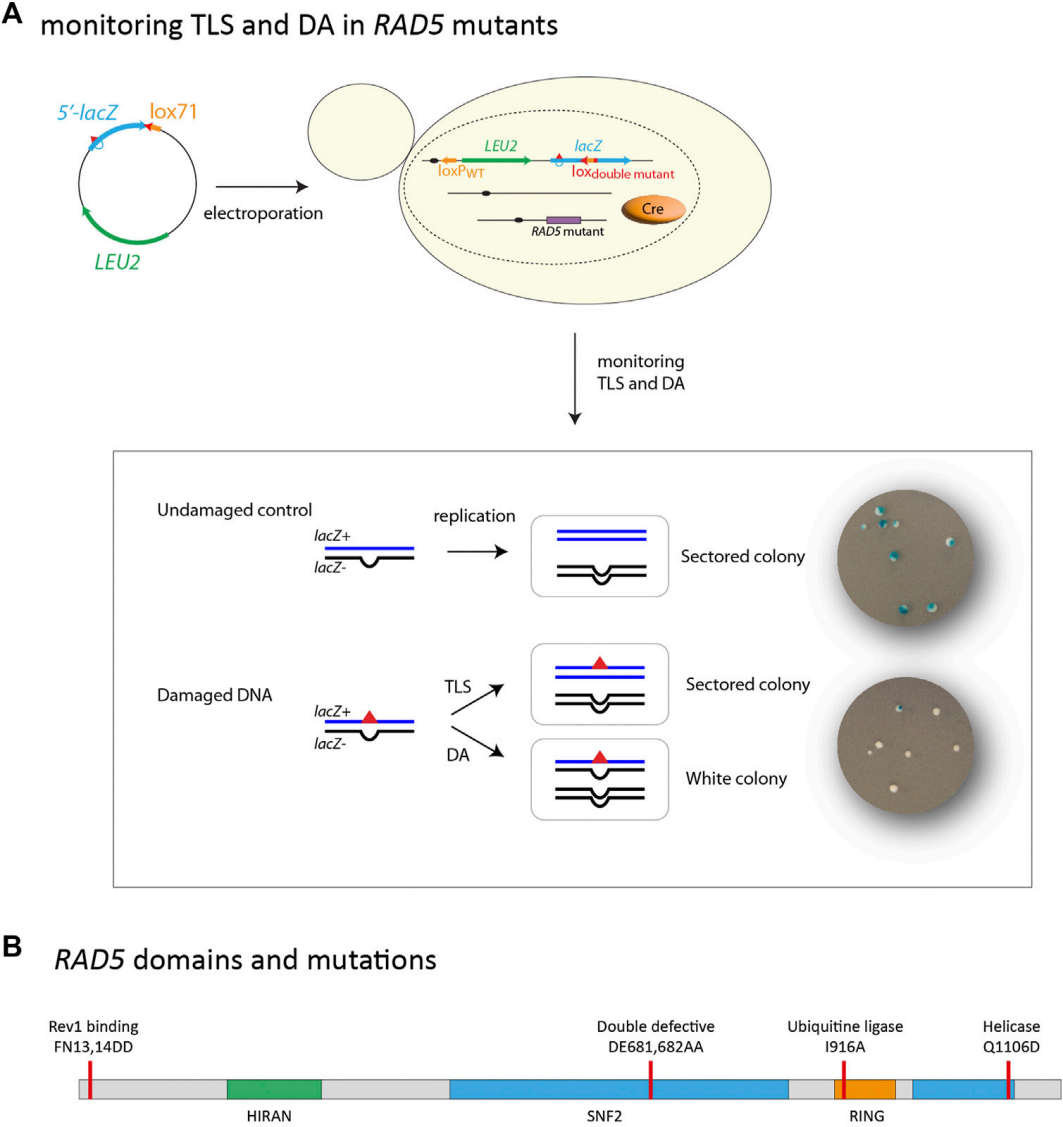
**(A)** outline of the integration system: A non-replicative plasmid containing a single lesion is integrated into one of the yeast chromosomes using Cre/lox site-specific recombination. The integrative vector carrying a selection marker (LEU2) and the 5′-end of the *lacZ* reporter gene containing a single lesion is introduced into a specific locus of the chromosome with the 3′-end of *lacZ*. Chromosomal integration of undamaged lac-/lac + heteroduplex constructs lead to sectored colonies on indicating media. Replication of the damaged heteroduplex yields a lac + event when the lesion is bypassed by TLS, whereas complementary strand replication yields a lac—event. Damage Avoidance (DA) events lead to two lac-events and therefore to the formation of white colonies. Lesion tolerance rates are calculated as the relative integration efficiencies of damaged vs. non-damaged vectors. **(B)** Schematic of *RAD5* gene highlighting the helicase domain as well as the RING ubiquitin E3 domain. Mutations used it this study are indicated. Mutation FN13,14DD affects the Rev1 binding site. Mutation DE681,682AA inactivates both the helicase and ubiquitin ligase activity. Mutation I916A inactivates the ubiquitin ligase activity. Mutation Q1106D inactivates the helicase activity.

As E3 ubiquitin ligase, Rad5 catalyzes PCNA polyubiquitination by bridging PCNA with the E2 ubiquitin-conjugating enzymes (Mms2-Ubc13) and accelerates ubiquitin transfer from the E2 to Ubi-PCNA. It also acts as a bridging factor to bring Ubc13 and Mms2 into contact with the Rad6/Rad18 complex, thereby providing a means to coordinate the distinct ubiquitin-conjugating activities of Rad6 and Ubc13/Mm2 and achieve specificity of the PCNA polyubiquitination (Ulrich and Jentsch, 2000; Carlile et al., 2009). As a DNA-dependent ATPase, Rad5 is a member of the DEAD box family of helicases. The two catalytic domains of Rad5 overlap: the RING E3 ligase domain responsible for E2 interaction is inserted between the conserved helicase motifs III and IV (Ulrich and Jentsch, 2000; Ulrich, 2003) (Figure 1B). It has been demonstrated that the Rad5 ATPase/helicase activity is not required for PCNA polyubiquitination (Choi et al., 2015). It has been shown that *in vitro*, the helicase domain of Rad5 has the capacity to catalyze the reversal of replication fork-like structures (Blastyák et al., 2007). Replication fork regression has been identified as a regulated response to replication stresses in eukaryotes, where it may provide protection to the stalled replication forks and facilitate template switching (Neelsen and Lopes, 2015). The role of replication fork reversal during replication stress in yeast cells seems to play a smaller role compared to higher eukaryotes (Blastyák et al., 2007). Chicken-foot structures in yeast have only been observed in checkpoint-defective mutants and are largely considered as pathological transactions at replication forks that have lost their replication capacity (Sogo et al., 2002; Cotta-Ramusino et al., 2005). It has also been suggested that Rad5 ATPase activity is important for DSB repair (Chen et al., 2005). Mutations of individual Rad5 helicase motifs show different effects (Chen et al., 2005; Minca and Kowalski, 2010; Ball et al., 2014; Ortiz-Bazán et al., 2014; Choi et al., 2015; Gallo et al., 2019). Therefore, the exact role of the helicase domain in damage bypass is still debated. We were able to show in this work, using a single lesion assay, that the helicase function of Rad5 does not participate in lesion bypass.

Rad5 also plays a structural role in the recruitment of TLS polymerases through physical interaction with Rev1 *via* its N-terminus (Xu et al., 2016). The fact that Rad5 is involved in both branches of DDT implies that it may play a role in the pathway choice and balance within DDT. However, we do not know precisely how the three domains of Rad5 (ubiquitin ligase, Rev1-binding, and helicase) could regulate the choice of the DNA Damage Tolerance pathway.

In this study we used domain-specific mutants to address the contribution of each of the Rad5 domains to the lesion tolerance. We demonstrate that the two critical functions of Rad5 during DNA damage tolerance are the activation of template switching through polyubiquitination of PCNA and the recruitment of TLS, and that loss of one of those functions can be compensated by increased usage of the other. We also show that the helicase activity does not play any role in lesion tolerance.

## Material and methods

### Strains and media

All strains used in the present study are derivative of strain EMY74.7 (Johnson et al., 1998) (MATa his3-Δ1 leu2-3,112 trp1-Δ ura3-Δ met25-Δ phr1-Δ rad14-Δ msh2Δ:hisG). In order to study tolerance events, all strains are deficient in repair mechanisms: nucleotide excision repair (*rad14*), photolyase (*phr1*), and mismatch repair system (*msh2*). Gene disruptions were achieved using PCR-mediated seamless gene deletion (Akada et al., 2006) or URAblaster (Alani et al., 1987) techniques. Rad5 point mutations were created using the delitto perfetto method (Storici and Resnick, 2006). All strains used in the study are listed in Table 1.

**TABLE 1:** Strains used in the study.

Strain	Relevant genotype (all strains are: MATa his3-Δ1 leu2-3,112 trp1-Δ ura3-Δ met25-Δ rad14-Δ phr1-Δ msh2Δ::hisG)
SC53	VI(167260–167265):: (lox66-3′lacZ-MET25/lag)
SC55	VI(167260–167265):: (lox66-3′lacZ-MET25/lead)
SC82	rev1-Δ VI(167260–167265):: (lox66-3′lacZ-MET25/lag)
SC83	rev1-Δ VI(167260–167265):: (lox66-3′lacZ-MET25/lead)
SC151	ubc13-Δ VI(167260–167265):: (lox66-3′lacZ-MET25/lag)
SC152	ubc13-Δ VI(167260–167265):: (lox66-3′lacZ-MET25/lead)
SC137	Rad5 (Q1106D) VI(167260–167265):: (lox66-3′lacZ-MET25/lag)
SC138	Rad5 (Q1106D) VI(167260–167265):: (lox66-3′lacZ-MET25/lead)
SC141	Rad5 (DE681,682AA) VI(167260–167265):: (lox66-3′lacZ-MET25/lag)
SC142	Rad5 (DE681,682AA) VI(167260–167265):: (lox66-3′lacZ-MET25/lead)
SC167	Rad5(I916A) VI(167260–167265):: (lox66-3′lacZ-MET25/lag)
SC168	Rad5(I916A) VI(167260–167265):: (lox66-3′lacZ-MET25/lead)
SC186	Rad5(FN13,14AA) VI(167260–167265):: (lox66-3′lacZ-MET25/lag)
SC187	Rad5(FN13,14AA) VI(167260–167265):: (lox66-3′lacZ-MET25/lead)
SC155	Rad5-Δ VI(167260–167265):: (lox66-3′lacZ-MET25/lag)
SC156	Rad5-Δ VI(167260–167265):: (lox66-3′lacZ-MET25/lead)
SC240	rev1-Δ Rad5 (DE681,682AA) VI(167260–167265):: (lox66-3′lacZ-MET25/lag)
SC241	rev1-Δ Rad5 (DE681,682AA) VI(167260–167265):: (lox66-3′lacZ-MET25/lead)
SC560	rev3-Δ::hisG Rad5(I916A) VI(167260–167265):: (lox66-3′lacZ-MET25/lag)
SC561	rev3-Δ::hisG Rad5(I916A) VI(167260–167265):: (lox66-3′lacZ-MET25/lead)
SC623	rev1-Δ Rad5(I916A) VI(167260–167265):: (lox66-3′lacZ-MET25/lag)
SC624	rev1-Δ Rad5(I916A) VI(167260–167265):: (lox66-3′lacZ-MET25/lead)

### Integration system

Integration of plasmids carrying (6‐4)TT/N2dG-AAF lesions (or control plasmids without lesion) and result analysis was performed as previously described (Maslowska et al., 2019). Lesion tolerance rates were calculated as the relative integration efficiencies of damaged vs. non-damaged vectors normalized by the transformation efficiency of a control plasmid (pRS413) in the same experiment. DA events are calculated by subtracting TLS events from the total lesion tolerance events.

All experiments were performed at least in triplicate. Graphs and statistical analysis were done using GraphPad Prism applying unpaired *t*-test. Bars represent the mean value ± s.d.

### Spotting assay

Overnight cultures of strains carrying Rad5 point mutations in YPD were adjusted to an OD_600_ value of 1. Volume of 10 μl from 10-fold serial dilutions of OD_600_-adjusted cultures were spotted on YPD agar plates containing different concentrations of 4-NQO (0 μM, 0,007 µM, 0,015 μM and 0.03 μM).

## Results and Discussion

### Rad5 is involved in damage avoidance through its ubiquitin ligase domain

Our group has recently developed an assay based on the insertion of a single replication-blocking lesion into a specific locus in the genome of a living yeast cell, which allows a phenotypical detection of TLS and DA events (Figure 1A) (Maslowska et al., 2019). Our system allows to monitor both error-free and mutagenic tolerance events, overcoming the limitations of assays measuring chromosomal mutagenesis after treatment with mutagenic agents which are blind to error-free events. In our previous study, we have demonstrated that inactivation of *ubc13* is compensated by a 10 fold increase in TLS usage (error-free and mutagenic events combined) at a (6–4)TT UV lesion, while other studies reported that *ubc13* inactivation led to a ∼2-fold increase in UV-induced mutagenesis (43), reflecting only the low fraction of mutagenic TLS events. Therefore, our method allows to provide more direct evidence for lesion bypass processes than previously used methods.

In the present work, we have used this assay to directly analyze the contribution of each of the Rad5 domains to both branches of the DNA damage tolerance, and determine their role in maintaining balance between TLS and DA. The use of site-specific DNA lesions provides more direct evidence for the role of Rad5 activities in different aspects of lesion bypass.

We have introduced a (6–4)TT photoproduct lesion (thymine-thymine pyrimidine (6–4)pyrimidone photoproduct), or a N2dG-AAF (N2-dG-Acetylaminofluorene) adduct in the genome of cells carrying mutations affecting different domains of the Rad5 protein (Figure 1B): i) an allele simultaneously deficient in Ubc13-binding and ATPase/helicase activity (DE681,682AA) named *RAD5DE* ^*ubi-helic*^ (Blastyák et al., 2007); ii) the Ubc13-binding RING domain (I916A) named *RAD5IA* ^*ubi*^ (Ulrich, 2003); iii) the helicase domain (Q1106D) named *RAD5QD* ^*helic*^ (Choi et al., 2015); iv) and the Rev1-binding domain (FN13,14AA) named *RAD5FN* ^*Rev1*^ (Xu et al., 2016); v) and a complete deletion of *rad5* gene. The results were compared to the parental strain expressing wild-type *RAD5* gene. In all strains we inactivated *rad14* to avoid repair of the lesion and focus on lesion tolerance mechanisms, and *msh2* to avoid repair of the strand marker (+2 nt loop) that allows to distinguish TLS from DA events.

**FIGURE 2:**
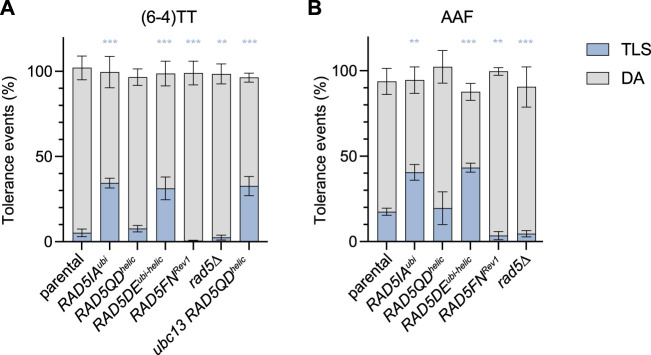
Partitioning of DDT pathways through (6—4)TT **(A)** and N2dG-AAF **(B)** lesions in domain-specific Rad5 mutants. Tolerance events represent the percentage of cells able to survive in presence of the integrated lesion compared to the lesion-free control. The data represent the average and standard deviation of at least three independent experiments. Unpaired *t*-test was performed to compare TLS values from the different mutants to the parental strain (*p < 0.05; **p < 0.005; ***p < 0.0005).

The mutation affecting Ubc13-binding (*RAD5IA* ^*ubi*^) led to a strong increase in TLS at both (6–4)TT photoproduct and N2dG-AAF lesions (Figures 2A,B). This increase in TLS is similar to the one we have previously observed in the absence of *ubc13* (Maslowska et al., 2019; Masłowska et al., 2022). It confirms that in the absence of PCNA poly-ubiquitination (either in the absence of ubc13, or by inactivation the ubiquitin ligase domain of Rad5), DA is reduced favoring TLS. We have previously described a competition between TLS and DA: in the absence of polyubiquitination of PCNA, DA is inhibited favoring TLS. However, it should be noted that in the absence of PCNA polyubiquitination there is still a proportion of cells surviving using a recombination pathway independent of PCNA ubiquitination that has previously been described as the salvage recombination pathway (Pfander et al., 2005). As shown in Figure 4, *RAD5IA* ^*ubi*^ also shows a high sensitivity to a global genotoxic stress such as 4NQO (4-Nitroquinoline-1-oxide) treatment, similar to the *ubc13∆* strain.

These data confirm the role of the ubiquitin-ligase function of Rad5 in promoting PCNA-ubiquitination and shows how it favors error-free lesion bypass.

### Rad5 interaction with Rev1 is required for Polζ-TLS

As we have shown previously, that TLS bypass of the (6–4)TT photoproduct relies almost exclusively on the TLS polymerases Rev1 and Pol ζ (Maslowska et al., 2019; Masłowska et al., 2022). The bypass of N2dG-AAF lesion is mostly dependent on Rev1 and Pol ζ, while a small part can be performed by pol η (Pagès et al., 2008; Masłowska et al., 2022). The *RAD5* allele unable to bind Rev1 (*RAD5FN* ^*Rev1*^) causes a severe decrease in the level of TLS at both (6–4)TT photoproduct and N2dG-AAF lesions (Figures 2A,B).

This indicates that the interaction of Rev1 with Rad5 is critical for its TLS activity *in vivo*. It has been shown previously that non-catalytic function of Rev1 in translesion synthesis and mutagenesis is mediated by its interaction with Rad5 (Kuang et al., 2013). Previous studies have also demonstrated that lack of the Rad5 N-terminal activity severely compromises spontaneous and DNA-damage-induced mutagenesis (Xu et al., 2016; Gallo et al., 2019).

**FIGURE 3:**
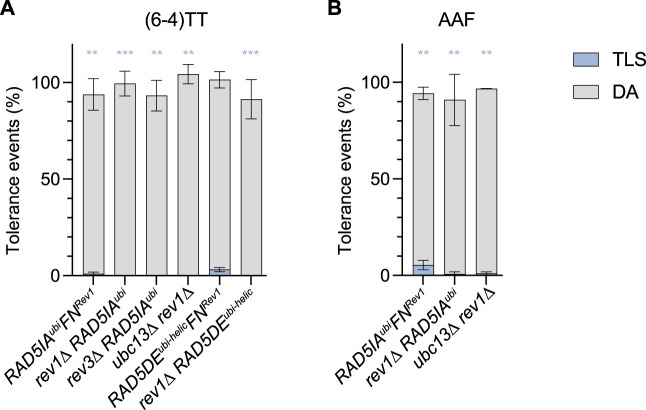
Role of Rev1 and Rad5-Rev1 interaction in the partitioning of DDT pathways through (6—4)TT **(A)** and N2dG-AAF **(B)** lesions. Tolerance events represent the percentage of cells able to survive in presence of the integrated lesion compared to the lesion-free control. The data represent the average and standard deviation of at least three independent experiments. Unpaired *t*-test was performed to compare TLS values from the different mutants to the parental strain (*p < 0.05; **p < 0.005; ***p < 0.0005).

For the (6–4)TT photoproduct, we confirmed that in the absence of PCNA polyubiquitination in mutants *RAD5DE* ^*ubi-helic*^ and *RAD5IA* ^*ubi*^, the strong increase in TLS (to a level >30%) was still exclusively due to Rev1-Pol ζ: as observed in Figure 3, the inactivation of *rev1* in the *ubc13*∆, *RAD5IA* ^*ubi*^, or *RAD5DE* ^*ubi-helic*^ mutants completely abolishes TLS (≤0.1%). Similarly, inactivation of Rev3 in the *RAD5IA* ^*ubi*^ also abolishes TLS. When preventing the recruitment of Rev1 by Rad5 (*RAD5FN* ^*Rev1*^) in the same mutants *RAD5IA* ^*ubi*^ and *RAD5DE* ^*ubi-helic*^, we also observed a strong decrease of TLS. However, unlike in the *rev1∆* strains, TLS is not completely abolished when combining default of PCNA polyubiquitination with *RAD5FN* ^*Rev1*^. It appears that when DA is inhibited, Rev1/Pol ζ can access the stalled fork and some TLS can occur despite the absence of recruitment of Rev1 by Rad5. However, this occurs at a much lower efficiency than when Rev1 is actively recruited by Rad5.

In response to 4NQO treatment, the *RAD5FN* ^*Rev1*^ show the same increased sensitivity as the *rev1∆* mutant, confirming the importance of Rad5 in recruiting Rev1. It remains however less sensitive than the mutant affecting PCNA polyubiquitination, reflecting the lesser role of TLS for survival over DA. When DA is abolished in the absence of PCNA polyubiquitination (strains *ubc13∆*, *RAD5IA* ^*ubi*^ or *RAD5DE* ^*ubi-helic*^), the further inactivation of rev1 either directly (*rev1∆* strain) or through the lack of recruitment by Rad5 (*RAD5FN* ^*Rev1*^) does not further increase the sensitivity to 4NQO, indicating again the minor role of TLS for survival.

**FIGURE 4:**
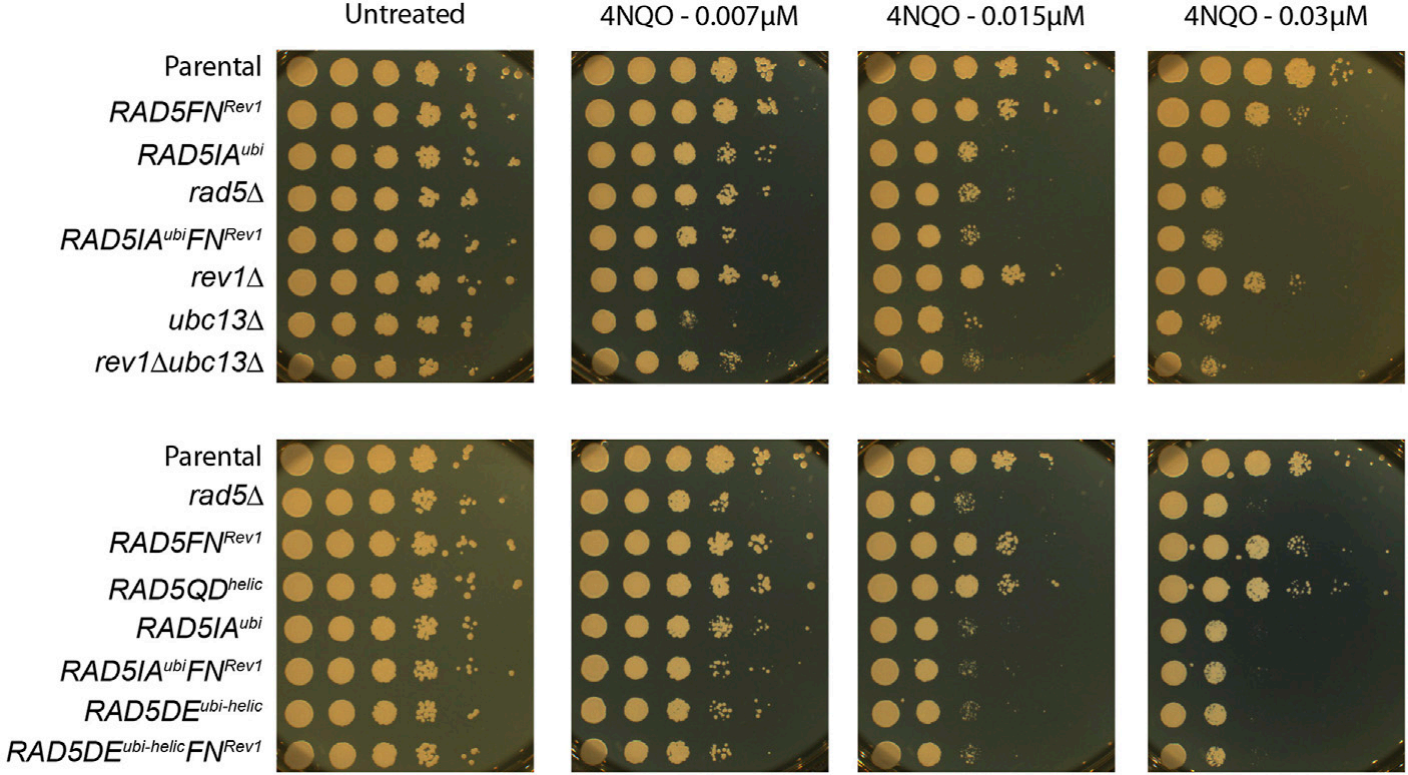
Sensitivity to 4NQO treatment of the different *RAD5* mutants.

While the complete deletion of *rad5* leads to an increased sensitivity to 4NQO (Figure 4), it does not lead to a drastic phenotype when monitoring the bypass of a single (6–4)TT photoproduct or N2dG-AAF lesions compared to WT *RAD5* (Figures 2A,B). We observed a significant decrease in TLS for the N2dG-AAF lesions, and a very moderate decrease for the (6‐4)TT photoproduct compared to the parental strain. It is important to note that in the *rad5*∆ strain, no polyubiquitination of PCNA occurs: we could therefore expect in these strains a strong increase of TLS as observed in the *ubc13*∆, *RAD5IA* ^*ubi*^, or *RAD5DE* ^*ubi-helic*^ mutants (Maslowska et al., 2019; Masłowska et al., 2022). However, due to the absence of Rad5 and its function of recruiting Rev1, TLS does not increase in this strain. Overall, the loss of DA due to the absence of PCNA-ubiquitination could not be compensated by an increase in TLS in the absence of Rev1 recruitment, and is therefore compensated by an increase in the salvage recombination pathway.

This illustrates the dual and opposite roles of Rad5 in lesion tolerance.

### Rad5 helicase function is not involved in damage tolerance

While the helicase function of Rad5 has been clearly evidenced *in vitro*, its functions *in vivo* remain controversial. Previous studies have shown that the helicase mutants are mildly sensitive to alkylating reagent-induced stress (Minca and Kowalski, 2010; Choi et al., 2015), but not to replication stress caused by nucleotide pool depletion (Gallo et al., 2019). However, those studies were done using different helicase mutants, therefore drawing clear conclusions from them is a difficult task. For that reason, the involvement of the helicase domain in lesion bypass remains unclear.

It is important to note that previous studies have considered the DE mutant solely as an ATPase defective strain and concluded as a role for the helicase function in DDT (Gangavarapu et al., 2006; Blastyák et al., 2007). However, it has since been showed that the DE mutation affects not only the helicase function, but also ubiquitin ligase activity due to reduced interaction with Ubc13 and PCNA (Ball et al., 2014; Choi et al., 2015). Therefore, the sensitivity that was observed for this mutant was most likely due to the polyubiquitination defect and not the helicase defect.

We have used a true separation of function mutation affecting solely the ATPase/helicase domain (Q1106D) of Rad5 (*RAD5QD* ^*helic*^). In this mutant, we did not observe any change of the level of TLS and DA at the (6–4)TT photoproduct and N2dG-AAF lesions (Figure 2A and B). It seems therefore that this function is not involved in the bypass of the tested lesions. One could wonder if the helicase function could act as a backup in the absence of DA. The levels of TLS and DA are the same in the mutant deficient for both helicase and ubiquitin ligase (*RAD5DE* ^*ubi-helic*^) as for the mutant deficient solely for the ubiquitin ligase (*RAD5IA* ^*ubi*^). Similarly, there is no difference between *ubc13∆* and *ubc13∆ RAD5QD* ^*helic*^ strains. These observations indicate that even in the absence of genuine DA that is dependent on PCNA polyubiquitination (*ubc13*∆ or *RAD5IA* ^*ubi*^), the helicase has no function in damage tolerance. We also generated a mutant deficient for both the ubiquitin-ligase and Rev1-binding domain (*RAD5IA* ^*ubi*^ *RAD5FN* ^*Rev*^), where only the helicase function of Rad5 remains (Figure 3A and B). These mutants show the same phenotype as a complete deletion of *RAD5*, confirming again that the helicase domain has no function in damage tolerance.

These results obtained with our two DNA lesions are compatible with previous observations from Gallo et al, (2019) who showed that in response to HU treatment, the helicase mutant had no effect on mutagenesis or survival. The absence of sensitivity of the *RAD5QD* ^*helic*^ mutant to 4NQO treatment (Figure 4) confirms that the helicase domain in not involved in lesion tolerance. Previous reports from Chen et al, (2005) have shown the involvement of the helicase function of Rad5 in double-strand break repair, a role that is independent from its ubiquitin ligase function. *In vitro* experiments have shown the involvement of the helicase domain in fork regression (Blastyák et al., 2007), a structure that could favor error-free lesion bypass. *In vitro* experiments have also suggested that Rad5 can facilitate strand invasion-dependent mechanisms in addition to fork regression for the template switching (Burkovics et al., 2014). It appears from our *in vivo* data and others (Chen et al., 2005; Gallo et al., 2019) that this is not a major pathway *in vivo*, at least for the tested lesions. While previous studies have suggested that the helicase function of Rad5 could contribute to lesion tolerance through fork regression, we show here that the helicase domain does not participate in the bypass of DNA lesions.

## Conclusion

From these data, we can draw the following model: Rad5 is recruited to the replication fork through its interaction with both Rad18 and PCNA (Ulrich and Jentsch, 2000), where it recruits Rev1 to allow TLS (Xu et al., 2016) and polyubiquitinates PCNA to allow DA. If Rad5 is unable to polyubiquitinate PCNA (*RAD5DE* ^*ubi-helic*^ or *RAD5IA* ^*ubi*^ mutant), it will recruit Rev1 and permit a high level of TLS as DA is inhibited.

If Rad5 is unable to interact with Rev1 (*RAD5FN* ^*Rev1*^ mutant), then Rev1-Polζ-TLS will be strongly reduced. A Rad5 protein defective for both its ubiquitin-ligase and Rev1-binding domains (*RAD5IA* ^*ubi*^, *FN* ^*Rev1*^) has a phenotype similar to a complete deletion of Rad5: it shows the same level of TLS and DA at the (6–4)TT photoproduct (compare Figures 2B, Figure 3), and similar sensitivity to 4NQO (Figure 4). In the absence of Rad5, Rev1 can still access the replication fork and perform TLS, but with a lower efficiency as it is not actively recruited by Rad5. It is worth noting that this double mutant (*RAD5IA* ^*ubi*^, *FN* ^*Rev1*^), despite the presence of a functional helicase domain show the same phenotype as a complete deletion of RAD5, showing that this function is not required for lesion tolerance.

In conclusion, we have shown that Rad5 plays two critical and opposite roles in lesion tolerance: i) through its ubiquitin ligase activity, Rad5 promotes error-free lesion bypass by damage avoidance, and ii) through its interaction with Rev1, it promotes Rev1/Pol ζ-dependent error-prone TLS. Our method allows to provide more direct evidence for lesion bypass processes than previously used methods. Therefore, using this method we were able to demonstrate that loss of one of the two main Rad5 functions can be compensated by increased usage of the other. Finally, we show that the helicase activity that has been suggested to favor error-free bypass by promoting fork regression does not play a role in the tolerance of isolated lesions.

A recent structural study by Shen et al, (2022) suggested that the Rad5 RING domain is mobile and has an autonomous function, consistent with our conclusions that the ubiquitin ligase and other activities of Rad5 contribute separately to replication stress tolerance. The same group demonstrated that Rad5 HIRAN domain mediates interactions with PCNA, contributing to its poly-ubiquitination by the RING domain, binds to DNA, and contributes to the Rad5-catalyzed replication fork regression (Shen et al., 2021). Therefore, the HIRAN domain may play a role in coordinating the multiple activities of Rad5 *in vivo*. It might be interesting in the future to investigate the role of the HIRAN domain in the recruitment of Rad5 at stressed replication forks. Overexpressing Rad5 results in aberrant template switching *via* HIRAN domain-mediated replication fork remodeling (Bryant et al., 2019). Mechanistic consequences of Rad5 overexpression might shed light on potential effect of human Rad5 homolog HLTF dysregulation impact on carcinogenesis, genome instability, and response to chemotherapy.

## Data Availability

The original contributions presented in the study are included in the article/supplementary material, further inquiries can be directed to the corresponding author.
